# Supplementary material for: The contribution of Earth observation technologies to the reporting obligations of the Habitats Directive and Natura 2000 network in a protected wetland
Source: PeerJ. 2018 Mar 21;6:e4540. doi: 10.7717/peerj.4540 (PMC5866716; doi:10.7717/peerj.4540)
Supplement: Appendix S1 — This appendix shows how the embryonic shifting dune (‘mobile dune’; 2110 habitat code) was moved since 2004 to 2014. We digitized the ‘mobile dune’ from aerial photography available for year 2004, 2008, 2010 and 2014. A shift of 66.5 meters over the last 10 years was estimated (Fig. S1). Fig. S2. Shape of the ‘mobile dune’ manually digitized from the aerial photography available for year 2004 (light green line), 2008 (red line), 2010 (dark green line) y 2014 (black line). [file peerj-06-4540-s001.pdf]

The contribution of Earth observation technologies to the reporting obligations of the Habitats Directive and Natura 2000 network in a protected wetland

Adrián Regos[1,2], Jesús Domínguez[2]

Appendix S1. Dynamics of the embryonic shifting dune.

This appendix shows how the embryonic shifting dune ('mobile dune'; 2110 habitat code) was moved since 2004 to 2014. We digitized the 'mobile dune' from aerial photography available for year 2004, 2008, 2010 y 2014. A shift of 66.5 meters over the last 10 years was estimated (Fig. S1).

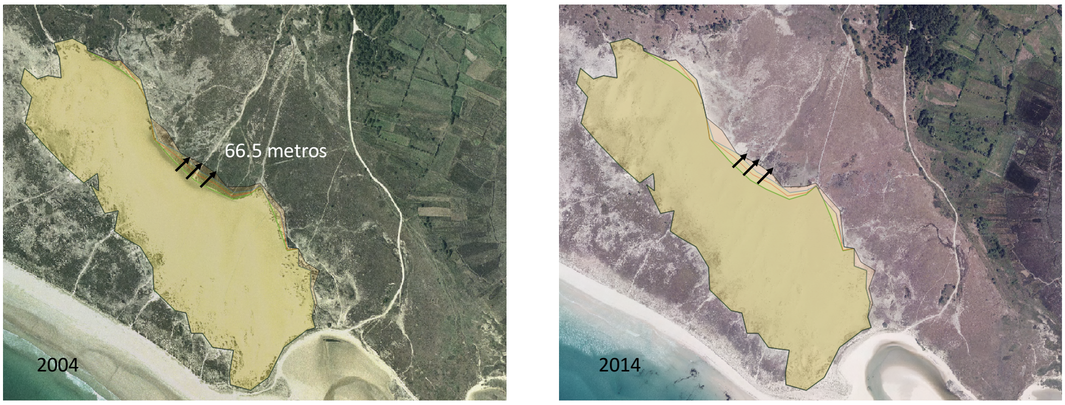

Fig. S2. Shape of the 'mobile dune' manually digitized from the aerial photography available for year 2004 (light green line), 2008 (red line), 2010 (dark green line) y 2014 (black line).
